# Safety and Molecular-Toxicological Implications of Cannabidiol-Rich Cannabis Extract and Methylsulfonylmethane Co-Administration

**DOI:** 10.3390/ijms21207808

**Published:** 2020-10-21

**Authors:** Kristy R. Kutanzi, Laura E. Ewing, Charles M. Skinner, Charles M. Quick, Stefanie Kennon-McGill, Mitchell R. McGill, Larry A. Walker, Mahmoud A. ElSohly, Bill J. Gurley, Igor Koturbash

**Affiliations:** 1 Department of Environmental and Occupational Health, University of Arkansas for Medical Sciences, Little Rock, AR 72205, USA; Kristy.kutanzi@gmail.com (K.R.K.); leewing@uams.edu (L.E.E.); cmskinner@uams.edu (C.M.S.); skennonmcgill@uams.edu (S.K.-M.); mmcgill@uams.edu (M.R.M.); 2 Department of Pharmacology and Toxicology, University of Arkansas for Medical Sciences, Little Rock, AR 72205, USA; 3 Department of Biochemistry, University of Arkansas for Medical Sciences, Little Rock, AR 72205, USA; 4 Center for Dietary Supplements Research, University of Arkansas for Medical Sciences, Little Rock, AR 72205, USA; bjgurley@olemiss.edu; 5 Department of Pathology, University of Arkansas for Medical Sciences, Little Rock, AR 72205, USA; quickcharlesm@uams.edu; 6 National Center for Natural Products Research, University of Mississippi, Oxford, MS 38677, USA; lwalker@olemiss.edu (L.A.W.); melsohly@olemiss.edu (M.A.E.); 7 ElSohly Laboratories, Inc. (ELI), Oxford, MS 38655, USA; 8 Department of Pharmaceutics and Drug Delivery, School of Pharmacy, University of Mississippi, Oxford, MS 38677, USA

**Keywords:** cannabidiol, cytochrome P450, dietary supplements, hemp extract, hepatotoxicity, herb-drug interaction, methylsulfonylmethane, safety, toxicity

## Abstract

Cannabidiol (CBD) is a biologically active, non-psychotropic component of *Cannabis sativa* whose popularity has grown exponentially in recent years. Besides a wealth of potential health benefits, ingestion of CBD poses risks for a number of side effects, of which hepatotoxicity and CBD/herb-drug interactions are of particular concern. Here, we investigated the interaction potential between the cannabidiol-rich cannabis extract (CRCE) and methylsulfonylmethane (MSM), a popular dietary supplement, in the mouse model. For this purpose, 8-week-old male C57BL6/J mice received MSM-containing water (80 mg/100 mL) ad libitum for 17 days. During the last three days of treatment, mice received three doses of CRCE administered in sesame oil via oral gavage (123 mg/kg/day). Administration of MSM alone did not result in any evidence of liver toxicity and did not induce expression of mouse cytochrome P450 (CYP) enzymes. Administration of CRCE did produce significant (*p* < 0.05) increases in *Cyp1a2*, *Cyp2b10*, *Cyp2c29*, *Cyp3a4*, *Cyp3a11*, *Cyp2c65*, and *Cyp2c66* messenger RNA, however, this effect was not amplified by MSM/CRCE co-treatment. Similarly, no evidence of liver toxicity was observed in MSM/CRCE dosed mice. In conclusion, short-term MSM/CRCE co-administration did not demonstrate any evidence of hepatotoxicity in the mouse model.

## 1. Introduction

Cannabidiol (CBD), a biologically active, non-psychotropic component of *Cannabis sativa*, has gained significant inroads into the US market over the last year with seemingly endless health claims positioning it as a proverbial panacea for treating stress and pain, boosting energy, enhancing circulation, and even curing arthritis and cancer [1,2,3]. CBD is a major component of Epidolex^®^, a drug indicated for the treatment of drug-resistant epileptic seizures associated with some rare pediatric syndromes [4,5,6]. Furthermore, recent studies indeed indicate significant potential CBD may have on social anxiety disorder and symptomatic improvement in schizophrenia (reviewed in [4,5,6]) Yet these promises may come at a cost, with a range of adverse side effects noted in vivo and among clinical trials, including diarrhea, vomiting, pneumonia, sedation, and somnolence [4,5,6]. Furthermore, cardiovascular, neurological, reproductive, embryo-fetal, gastrointestinal, and liver toxicity have been documented subsequent to CBD use at higher doses [5,7,8,9,10,11,12,13,14]. To add to these complications, numerous studies have demonstrated that CBD possesses a significant potential for drug interactions [15,16,17,18,19].

Among side effects associated with higher dose CBD ingestion, the risk for hepatotoxicity is a major concern [20]. For instance, plasma levels of liver aminotransferases were greater than three times the upper limit of normal in patients taking valproate while being treated with Epidiolex^®^, a purified CBD-containing drug used to treat some rare forms of pediatric refractory epilepsy [16,17,21]. Recent findings have shown that cannabidiol-rich cannabis extract (CRCE) given concomitantly with acetaminophen (APAP), one of the most common over-the-counter medications, exacerbates CBD hepatotoxicity, leading to sinusoidal obstruction syndrome-like liver injury and mortality in the mouse model [15]. These effects were associated with ingestion of high (therapeutic range) doses of CBD, however, the widespread and relatively indiscriminate distribution of CBD-containing products in the supplement market, and the potential inter-individual variability in response to CBD raises concerns as to co-ingestion of CBD and CBD-containing products with prescription and nonprescription drugs.

Dietary supplements (DSs) represent a large group of food items whose regulatory landscape is defined by the Dietary Supplement Health and Education Act (DSHEA 1994). According to DSHEA, pre-market safety assessments for DSs are not required and, while most DSs pose no serious concerns for toxicity, rare cases of herb-induced hepato- and cardiotoxicity as well as clinically relevant herb-drug interactions argue for a more cautious approach to assessing DS safety [22,23,24].

According to recent polls, up to 70% of U.S. adults took at least one DS within the last 12 months. While it is difficult to realistically assess the current market for CBD and CBD-containing products, their increased popularity suggests that the fraction of U.S. consumers ingesting these products is substantial. Therefore, the probability for concomitant ingestion of CBD with other DSs is very likely, and the potential for CBD–DS interactions necessitates thorough investigation.

In this regard, methylsulfonylmethane (MSM), a natural organosulfur compound, is of particular interest. MSM is found in a wide range of foods, including certain vegetables, fruits, grains, and beverages. Also known as dimethyl sulfone, MSM has become a popular DS, largely for its anti-oxidative and anti-inflammatory properties with some studies indicating that ~10% of supplement users regularly ingest MSM [25]. Numerous patents claiming positive health benefits ranging from stress and pain relief to increased energy and metabolism, enhanced circulation, and improved wound healing [26,27,28,29], along with publications showing potential clinical benefit in arthritis and other inflammatory disorders [30,31], have been exploited in marketing of MSM as a popular supplement.

MSM is readily absorbed in the small intestine of both rodents and humans and is characterized by rapid distribution throughout tissues, including the small intestine and liver [32]. Studies indicate that MSM exerts its antioxidant and free-radical scavenging properties by targeting the transcriptional activity of nuclear factor kappa-light-chain enhancer of activated B cells (NF-kB), signal transducers and activators of transcription (STAT), p53, and nuclear factor (erythroid-derived 2)-like 2 (Nrf2) (reviewed in [25]). However, whether or not MSM is capable of affecting cytochrome P450 (CYP) enzymes in the liver remains unknown. Furthermore, while numerous studies indicate the safety of MSM even at high doses, the potential for MSM-drug/herb interactions remains unexplored.

Therefore, the aim of this study was to investigate whether MSM administration affects the expression of major CYP isoforms and if concurrent use of MSM with CRCE results in MSM–CRCE interactions and/or sensitization to CRCE-induced liver injury.

## 2. Results

### 2.1. Phytocannabinoid Characterization of CRCE

CRCE phytochemical characterization results are presented in Table 1. This phytocannabinoid composition is comparable to those of CRCEs used in previous studies, as well as in products currently on the U. S. market [13,14,15,33]. Other measurements were as follows: loss on drying—0.32%; heavy metals: lead, mercury, cadmium, and arsenic—not detected; aflatoxins: AFB_1_, AFB_2_, AGF_1_, and AFG_2_—not detected; *Escherichia coli*—absent; *Salmonella*—absent; Total Aerobic Microbial Count (TAMC)—<10 cfu/g; Total Yeast and Mold Count (TYMC)—<10 cfu/g; all of which are below USP acceptable levels for non-sterile oral preparations.

### 2.2. Anatomical Examination and Physiological Parameters

**Figure 1:**
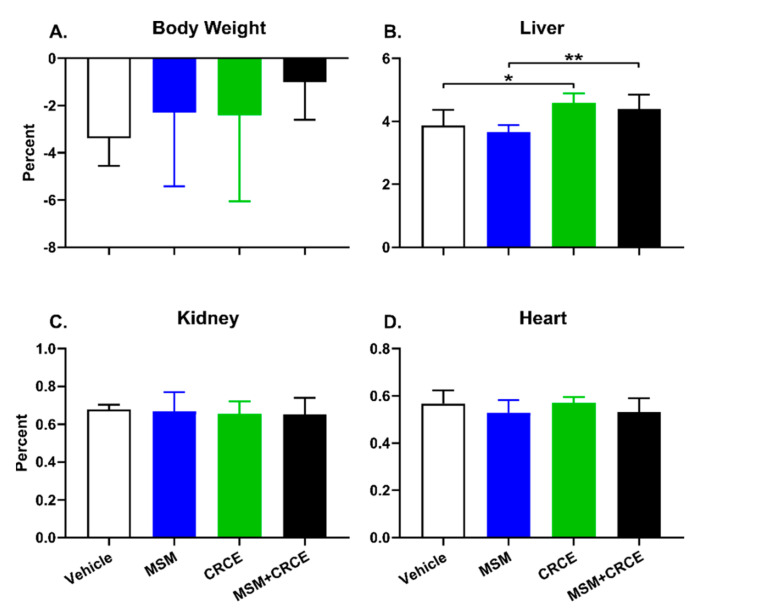
Physiological parameters in response to methylsulfonylmethane (MSM), CRCE, or MSM/CRCE administration in C57BL6/J mice. (**A**) Body weight change, (**B**) liver-to-body weight, (**C**) kidney-to-body weight, and (**D**) heart-to-body weight ratios. Data are presented as mean ± SD (*n* = 6–8). * indicates a significant difference (*p* < 0.05), and ** indicates a significant difference (*p* < 0.01), as calculated with a two-way ANOVA and Bonferroni post-hoc test. MSM—methylsulfonylmethane, CRCE—cannabidiol-rich cannabis extract.

Throughout the study there were no signs of overt toxicity. Gross anatomical examination upon termination found no visual abnormalities in the liver, kidney, or heart. Although there were no statistically significant differences in body weight between groups, liver-to-body weight ratios, in agreement with previous studies, were increased in mice treated with CRCE or MSM/CRCE (Figure 1A,B). No significant differences in kidney- and heart-to-body weight ratios were observed (Figure 1C,D).

No significant differences were observed in food or water consumption between any of the groups throughout the experiment (data not shown).

### 2.3. Histological Findings

**Figure 2:**
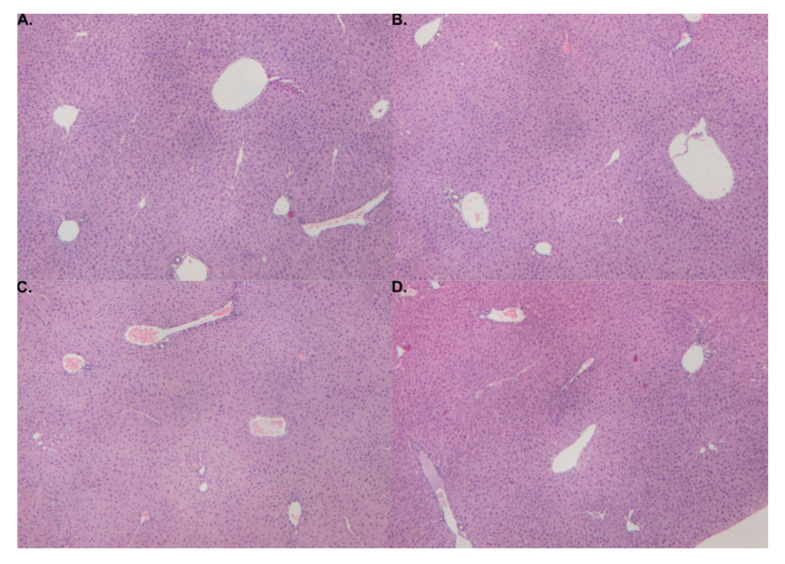
Effects of MSM, CRCE, or MSM/CRCE on liver morphology. (**A**) vehicle (sesame oil); (**B**) MSM; (**C**) CRCE; (**D**) MSM/CRCE. Magnification: ×40. MSM—methylsulfonylmethane, CRCE—cannabidiol-rich cannabis extract.

Histopathological assessment identified no evidence of necrotic, apoptotic, hemorrhagic, or inflammatory events in experimental mice (Figure 2).

### 2.4. Clinical Biochemistry

**Figure 3:**
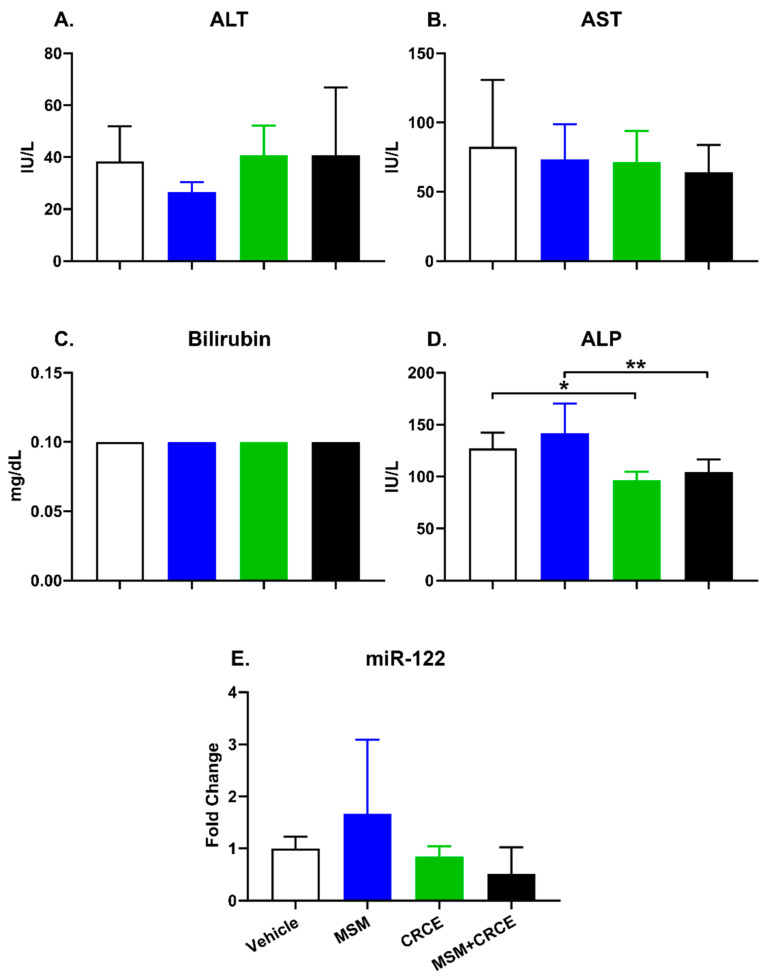
Effects of MSM, CRCE, or MSM/CRCE on plasma biomarkers of liver injury. (**A**) alanine aminotransferase, ALT; (**B**) aspartate aminotransferase, AST; (**C**) total bilirubin; (**D**) alkaline phosphatase (ALP); (**E**) miR-122. Data are presented as mean ± SD (*n* = 6–8). * indicates a significant difference (*p* < 0.05), and ** indicates a significant difference (*p* < 0.01), as calculated with a two-way ANOVA and Bonferroni post-hoc test. MSM—methylsulfonylmethane, CRCE—cannabidiol-rich cannabis extract.

Analysis of clinical biomarkers of liver injury revealed no MSM- or CRCE- associated elevations in plasma alanine- and aspartate-aminotransferases (ALT and AST, respectively), nor in total bilirubin (Figure 3A–C). Decreased alkaline phosphatase (ALP) levels were noted in the plasma of CRCE-treated animals, with no significant difference observed between mice treated with MSM/CRCE compared to CRCE alone (Figure 3D). Also, there were no measurable differences in circulating liver-specific miR-122, a novel sensitive biomarker of liver injury (Figure 3E).

### 2.5. Cytochrome P450 Expression

**Figure 4:**
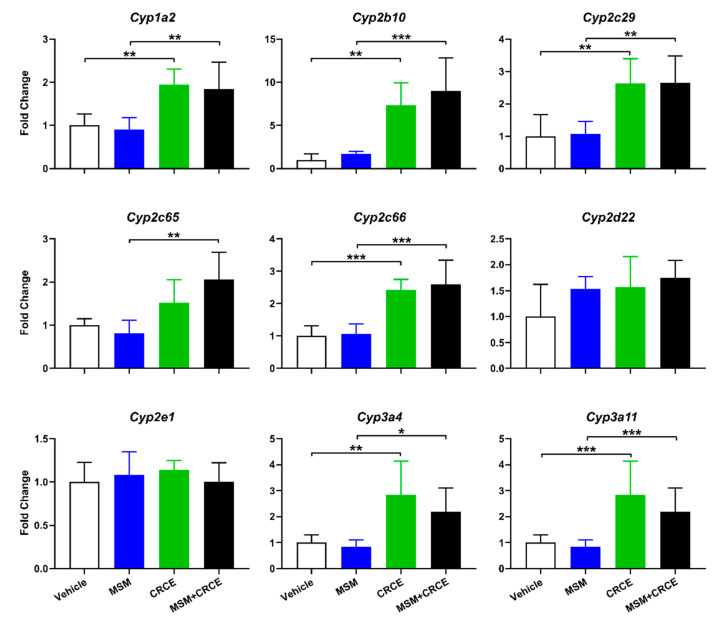
Effects of administration of MSM, CRCE, or MSM/CRCE on intrahepatic expression of cytochrome P450s. Livers were collected 24 h after the last gavage and gene expression was measured using the quantitative real-time (qRT) PCR. Data was analyzed by two-way ANOVA with Bonferroni post-test and presented as mean ± SD fold change from vehicle (*n* = 6–8), with * as *p* < 0.05; ** as *p* < 0.01, and *** as *p* < 0.001. MSM—methylsulfonylmethane, CRCE—cannabidiol-rich cannabis extract.

Many compounds, regardless of their effect on liver morphology, can have major implications on liver function. To determine if MSM alone or in combination with CRCE could affect the expression of liver drug metabolizing enzymes at administered doses, we evaluated a panel of mouse CYP isoforms (Figure 4).

MSM alone did not affect the hepatic expression of any of the nine investigated CYPs. However, consistent with previous findings, administration of CRCE for three consecutive days increased mRNA levels for seven CYP genes, with *Cyp2b10* being most affected (~eight-fold increase) (Figure 4). Interestingly, no induction of *Cyp2e1* was observed, an isoform previously reported to be up-regulated shortly after CRCE administration [13]. The expression of *Cyp2d22*, an isoform considered non-inducible, remained unaffected.

Co-administration of MSM with CRCE produced no additional changes in mRNA, as no significant differences were observed between the CRCE and MSM/CRCE experimental groups. These findings suggest that several key Phase I metabolic pathways in the liver may be compromised by CRCE, however, MSM itself does not affect hepatic CYP expression nor does it influence CRCE-associated CYP induction.

### 2.6. Glutathione Measurement

Finally, we evaluated the effect of MSM/CRCE co-administration on the synthesis of hepatic glutathione. The toxicity of xenobiotics is buffered by glutathione, which plays a critical role in protecting cells from both oxidative damage and electrophilic xenobiotic metabolites. Previous studies have indicated that MSM can increase hepatic glutathione synthesis, while administration of high CRCE doses were associated with pro-oxidative responses [13,34]. Therefore, it was important to evaluate how co-administration of CRCE with MSM might affect glutathione synthesis.

**Figure 5:**
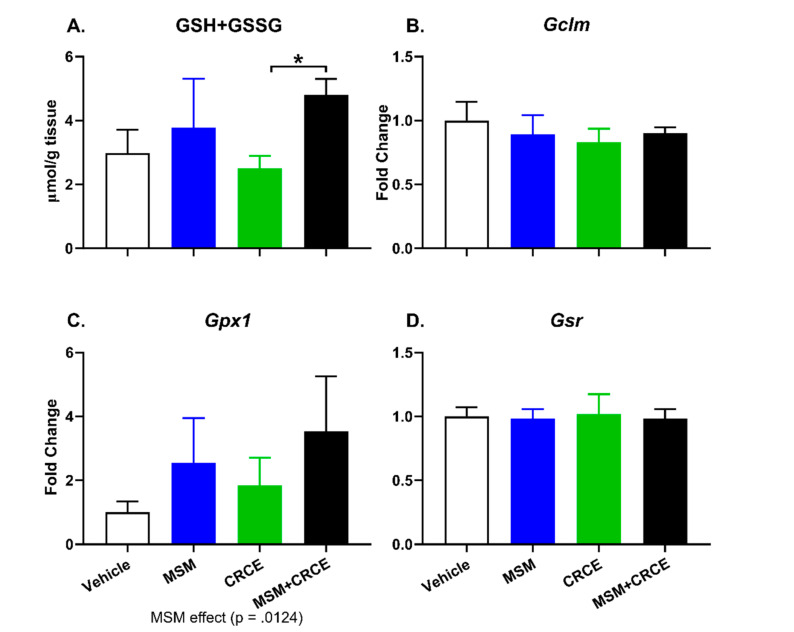
Effects of MSM, CRCE, or MSM/CRCE on intrahepatic synthesis of glutathione and the expression of genes associated with the synthesis of glutathione. (**A**) Intrahepatic concentrations of total glutathione; (**B**–**D**) mRNA levels of (**B**) *Gclm*, (**C**) *Gpx1*, and (**D**) *Gsr* in the mouse livers 24 h after the last dose of CRCE. Gene expression was measured using quantitative real-time (qRT) PCR. Data was analyzed by two-way ANOVA with Bonferroni post-test and are presented as mean ± SD fold change from vehicle (*n* = 6–8), with * as *p* < 0.05.

Consistent with previous findings, administration of MSM resulted in modest increases in glutathione, while CRCE alone produced slight reductions in total intrahepatic glutathione (Figure 5A). Furthermore, total glutathione was nearly two-fold higher in MSM/CRCE mice compared to CRCE alone, suggesting the CRCE did not negatively regulate MSM-associated induction of intrahepatic glutathione synthesis. Also, several key enzymes involved in different stages of glutathione synthesis (e.g., *Gclm*, *Gpx1*, and *Gsr*) were unaffected by concomitant ingestion of MSM and CRCE (Figure 5B–D).

## 3. Discussion

CBD and CBD-containing products are currently on the rise throughout healthcare markets worldwide. Despite its high popularity among consumers, knowledge regarding the negative health effects of CBD-containing products remains limited. Accumulating evidence, however, indicates that CBD, when administered at clinically relevant doses, possesses significant potential for hepatotoxicity as well as for interactions with various conventional medications. In our previous studies, we have investigated the hepatotoxic potential of CRCE, either alone or when co-administered with another potent hepatotoxicant—APAP [13,15]. Therefore, the present study was designed to explore the potential interaction between CRCE and MSM—a popular DS on the U. S. market—that, due to their widespread usage, may overlap in the treatment of various ailments.

Ingestion of MSM itself resulted in no overt toxicological responses, including hepatotoxicity, among experimental mice. This finding is in line with previously conducted studies as well as its Generally Recognized as Safe (GRAS) status attained in 2007 [35]. Similarly, there was no histopathological evidence of liver injury in mice gavaged with CRCE, nor with the combination of MSM and CRCE as determined by plasma aminotransferases, bilirubin, and miR-122.

At the same time, administration of CRCE or CRCE/MSM caused a significant increase in the liver-to-body weight ratio in mice. This corroborated the findings of other animal studies examining the effects of CBD or CRCE alone [12,13], or in combination with other hepatotoxicants [15]. Xenobiotic-induced increase in liver weight and liver-to-body weight ratio is not a rare finding in rodent models and is not necessarily a prerequisite of liver injury (especially, when other more reliable markers of liver injury are absent) and may be considered an adaptive response. Such adaptive responses may, in turn, be triggered by robust induction of drug metabolizing enzymes, especially Phase I enzymes such as CYPs [36]. Indeed, in this study, CRCE caused a significant increase in the mRNA levels of seven out of nine mouse CYP enzymes. For some like *Cyp2b10*, which is homologous to human CYP2B6, the increase was substantial, reaching nearly 10-fold. This finding is of particular importance, since tissue harvest occurred 24 h after the last CRCE dose. Taking into account the higher metabolic rates of mice, this suggests CRCE has prolonged effects on hepatic drug metabolizing enzyme activity. Such CYP-associated increases in liver weight are usually adaptive responses, serving a role of metabolism enhancement and detoxification, and often resolve shortly after xenobiotic exposure ceases. The prolonged ingestion of a given xenobiotic may, however, lead to decompensation of adaptive responses and result in hepatotoxicity. Interestingly, most clinical cases of hepatotoxicity associated with the CBD in the form of the drug Epidiolex^®^ were due to its prolonged intake and rarely occurred during the first weeks of treatment [21].

Out of seven CYP enzymes whose expression was induced by CRCE, of particular interest is *Cyp2b10*, a mouse enzyme homologous to human CYP2B6. In CRCE-treated groups, its expression reached nearly 8–10-fold compared to vehicle-treated or MSM-only animals. This data is in agreement with findings within the B6C3F_1_ mouse strain, in which *Cyp2b10* was the most up-regulated CYP gene after both single and repeated dosing with CRCE [13]. This CYP isoform is known to play a key role in the metabolism of prescription drugs, such as cyclophosphamide, ketamine, and bupropion. Furthermore, CYP2B6 together with CYP3A4 are central to the metabolism of clobazam, a common anti-seizure medication used in the treatment of many forms of epilepsy. Here, we demonstrate that CRCE, besides eliciting a robust upregulation of *Cyp2b10* mRNA, increased expression of two CYP3A4 mouse homologs*—Cyp3a4* and *Cyp3a11*. Clinical trials confirm this finding, demonstrating higher serum concentrations of *N*-desmethylclobazam, the active metabolite of clobazam, when the latter is co-administered with CBD (Epidiolex^®^) [24]. Though this elevation of N-desmethylclobazam may also be attributable to inhibition of CYP2C19, contributions of CYP3A and CYP2B6 induction have also been recognized [37,38]. This ability to serve as a substrate for multiple CYPs suggests that CBD-containing products may have a significant interaction potential with other drugs besides clobazam.

MSM is rapidly absorbed from the gastrointestinal tracts of both humans and rodents [39]. The exact metabolic fate of MSM is not fully understood, however, its tissue distribution and liver accumulation as well as formation by hepatic microsomes have been reported [40]. Studies in rats further demonstrate that up to 80% of MSM is excreted unchanged [41]. Here, we demonstrated that MSM caused no measurable changes in the expression of a panel of mouse hepatic Cyps. Furthermore, pre-treatment with MSM for two weeks did not affect CRCE-associated CYP induction, suggesting a rather inert nature of MSM in the mouse liver.

Despite the liver’s seemingly minor role in MSM clearance, an indirect potential for an MSM/CRCE interaction cannot be dismissed. For instance, a recent report indicated an important role *Cyp2c29*, another CYP affected by CRCE in this study, plays in liver injury [42]. This homolog of human CYP2C8 and CYP2C9 was found to be continuously down-regulated during liver injury. In contrast, reversal of *Cyp2c29* expression in APAP- and CCl_4_-induced hepatotoxicity (two common models of liver injury), resulted in suppression of pro-inflammatory signaling and cytokine production by targeting NF-kB pathway [42]. Interestingly, similar anti-inflammatory effects associated with NF-kB inhibition were reported for MSM [43,44]. These findings may have a significant therapeutic value and further investigations of the MSM/CRCE interaction potential and its effects on NF-kB signaling pathway are clearly warranted.

Previous studies have demonstrated that high doses of CBD/CRCE can deplete intrahepatic glutathione and its synthesis [13,15]. The latter plays a critical role in protecting cells from oxidative damage and the toxicity of xenobiotic electrophiles by maintaining redox homeostasis. MSM has been previously reported to effectively modulate synthesis of glutathione in experimental models [34]. The exact mechanism(s) underlying this modulatory effect remain unknown, but by serving as an alternate source of sulfate for the sulfation and detoxification of various compounds, MSM may spare cysteine for the synthesis of glutathione [45,46,47]. Therefore, it is critical to understand whether or not the ability of MSM to regulate glutathione will be hampered by concurrent ingestion of other DSs, particularly the popular glutathione-depleting CBD-containing products. Importantly, we demonstrate that mice treated with MSM and subsequently challenged with CRCE exhibited a nearly two-fold increase in hepatic glutathione levels, compared to CRCE alone. Furthermore, the potential of MSM to mitigate toxin-induced liver damage, such as with carbon tetrachloride [48], paraquat [45], or APAP [46], suggests that MSM can serve as a valuable hepatoprotective agent, including in cases of CBD/CRCE-induced liver injury.

In conclusion, we report that concurrent short-term administration of MSM with CRCE is not hepatotoxic in an experimental mouse model. Furthermore, the potential for an MSM/CRCE interaction appears low based upon the lack of CYP modulation by MSM. Lastly, MSM’s ability to enhance hepatic glutathione synthesis was not affected by CRCE, a finding suggestive of its capability for mitigating CBD/CRCE-induced hepatotoxicity. Future studies are warranted to explore this potential, as well as the nature of long-term co-administration of CRCE with other entities.

## 4. Materials and Methods

### 4.1. CRCE Extract Characterization, Dosing Solution, and Dose Calculations

The product was prepared by extracting a CBD-rich, THC-poor *Cannabis sativa* L. chemovar (commonly referred to as hemp) (5.61% of CBD and 0.2% THC *w/w*) using hexane as a solvent. The ratio of CBD to THC in the extract was the same as in the plant material (28:1). The extract was evaporated to dryness and held at 80 °C for several hours in order to effect complete decarboxylation of the phytocannabinoids.

CRCE doses were calculated to deliver the required amount of CBD. CRCE diluted in sesame seed oil served as the final oral gavage solution. Allometric scaling for CBD mouse equivalent doses (MEDs) was determined per the recommendation of Wojcikowski and Gobe [49], which, in turn, is based upon an FDA Industry Guidance [50]. MEDs were based upon the recommended human maintenance dosage of purified CBD (Epidiolex^®^) as well as our previous experience with CRCE in various animal models [13,14,15]. The quantity of CBD administered to a 25 g mouse (average animal weight in this study) was 10 mg/kg/day × 12.3 (mouse allometric scaling factor) to yield a total CBD dose of 123 mg/kg/day delivered in sesame seed oil at a total volume of 300 µL. Control mice received 300 µL of sesame seed oil.

### 4.2. Animals

Male C57BL6/J (7-weeks of age) were purchased from Jackson Labs (Bar Harbor, MA, USA). Animals were acclimated for 1 week prior to study initiation and randomized into cohorts of vehicle (sesame seed oil) and experimental groups: MSM, CRCE, and MSM/CRCE (*n* = 6 per group, except for the MSM/CRCE group which contained 8 animals). Each animal was individually identified with an ear tag. Mice were housed 3 per cage (4 animals per cage for the MSM/CRCE group) in polycarbonate cages in an appropriate animal room at the University of Arkansas for Medical Sciences (UAMS) Division of Laboratory Medicine from arrival until euthanasia. Room temperature was maintained at 19–22 °C with a relative humidity of 55–70%. Automatic light controls were set to provide fluorescent lighting for a 12 h photoperiod (07:00–19:00 for light phase). All procedures were approved by the UAMS Institutional Animal Care and Use Committee (protocol number: AUP #3902), and all personnel followed appropriate safety precautions.

Mice received MSM (Bergstrom Nutrition, Vancouver, WA, USA) in drinking water (80 mg/100 mL) ad libitum for 17 days. The dose of MSM chosen for this study was based on previous reports [51]. Water intake was measured frequently to ensure dosage of MSM did not differ significantly between animals. Animals in the CRCE groups were gavaged with 123 mg/kg/day CBD (MED of 10/mg/kg/day, a commonly used dose of CBD in clinical settings) on Monday, Tuesday, and Wednesday of Week 3 (days 15 through 17) at 08:00. All animals were scheduled to be terminated 24 h after the last gavage treatment of the CRCE groups. Pelleted rodent diet was available ad libitum except for an overnight fasting period prior to necropsy.

Mice were anesthetized with isoflurane and terminal bleeding was performed by a trained animal technician. Anesthetized animals then underwent cervical dislocation to ensure death and tissues were collected immediately. Animal body, liver, heart and kidney weights were recorded at the time of necropsy. Livers were excised, 1-mm sections were obtained from the left and right lateral lobes, and placed in a tissue cassette for further histological evaluation. The remaining liver tissue was snap-frozen in liquid nitrogen and stored at −80 °C for further molecular and biochemical analysis.

### 4.3. Histopathological Assessment

Liver sections were fixed in 4% formalin for 24 h, then briefly rinsed in PBS and stored in 70% ethanol for 24 h. Livers were then processed at the UAMS Pathology Core Facility, stained with hematoxylin and eosin, and evaluated by a board-certified pathologist in a blinded fashion.

For histologic assessment purposes, each liver was represented by two sections obtained from different locations within the liver. Each section was initially evaluated at magnifications of ×40 and ×100. Sections were further evaluated at ×200 and ×400 to check for the presence of mitotic figures, necrotic foci, and apoptotic bodies.

### 4.4. Blood Sampling and Clinical Biochemistry

To measure the effects of CBD extract on a panel of liver enzymes characteristic for drug-induced liver injury, blood was collected from the retroorbital plexus with a heparinized micro-hematocrit capillary tube (Fisher Scientific, Pittsburg, PA, USA) and placed into a 1.1-mL Z-gel microtube (Sarstedt, Newton, NC, USA). Tubes were kept on ice and centrifuged at 10,000 rpm for 20 min; serum samples were then immediately aliquoted and delivered to the Veterinary Diagnostic Laboratory at the Arkansas Livestock and Poultry Commission (Little Rock, AR, USA) on dry ice where samples were processed the same day.

### 4.5. miR-122 Analysis

miR-122 levels were measured in serum by qRT-PCR as previously described [52]. Briefly, RT reactions, pre-amplification, and qRT-PCR were carried out for each sample. Two-step RT reactions were performed in a 15 µL-RT reaction using the TaqMan miRNA Reverse Transcription Kit (Life Technologies, Foster City, CA, USA). qRT-PCR reactions were run in 10 µL composed of 5 µL TaqMan Universal PCR Master Mix (2×), 0.5 µL water, 0.5 µL Taqman miRNA qPCR assay (20×) and 4 µL diluted Pre-Amp products on a ViiA7 qRT-PCR thermocycler at 95 °C for 10 min, 40 cycles at 95 °C for 15 s and 60 °C for 1 min, and held at 4 °C. The miRNA expression values were normalized to the internal control *U6* and expressed as fold change according to the ΔΔC_t_ method.

### 4.6. Gene Expression

Total RNA was extracted from liver tissue using the RNeasy Mini Kit (QIAGEN, Germantown, MD, USA) according to the manufacturer’s protocol. Following purification, 1000 ng were reverse transcribed with the High Capacity cDNA Reverse Transcription Kit following the manufacturer’s instructions (Thermo Fisher, Waltham, MA, USA). Primers were added at a final concentration of 5 µM. The list of assays used in this study can be found in Table S1. Gene expression values were normalized to the internal control gene *18S* and expressed as fold change according to the ΔΔC_t_ method.

### 4.7. Glutathione Measurement

Total glutathione (GSH + GSSG) in the liver was measured using a modified Tietze assay as previously described [53]. Briefly, liver tissues were homogenized in 3% sulfosalicylic acid using a bead-ruptor homogenizer. Aliquots were then diluted 50-fold in 0.01 M HCl, centrifuged, and the deproteinated supernatants further diluted 26-fold in 100 mM potassium phosphate buffer. Total glutathione was measured in the samples using the Tietze cycling method with glutathione reductase and 5,5-dithio-bis-(2-nitrobenzoic acid).

### 4.8. Statistical Analysis

All statistical analyses were performed with the Graphpad Prism 6 software (Graphpad Software. San Diego, CA, USA). Groups were compared with a two-way ANOVA followed by a Bonferroni multiple comparison test when appropriate. An adjusted *p*-value of <0.05 was considered significant.

## Figures and Tables

**Table 1:** Phytocannabinoid characterization of cannabidiol-rich cannabis extract (CRCE).

Phytoconstituent	Levels, %
Cannabidiol	57.9
Cannabichromene	2.03
Δ^9^-tetrahydrocannabinol	1.69
Cannabigerol	1.07
Δ^8^-tetrahydrocannabinol	<0.01
Tetrahydrocannabivarin	<0.01
Residual Solvent	<0.05

## Supplementary Materials

Supplementary materials can be found at https://www.mdpi.com/1422-0067/21/20/7808/s1.

## Abbreviations

ALT	Alkaline phosphatase
ALT	Alanine aminotransferase
AST	Aspartate aminotransferase
CBD	Cannabidiol
CRCE	Cannabidiol-rich cannabis extract
CYP	Cytochrome P450 enzymes
DS	Dietary supplements
GSH	Glutathione
MED	Mouse equivalent dose
MSM	Methylsulfonylmethane
NEM	*N*-ethylmaleimide
PCR	Polymerase chain reaction

## References

[1] Alves P., Amaral C., Teixeira N., Correia-da-Silva G. (2020). Cannabis sativa: Much more beyond Δ9-tetrahydrocannabinol. Pharmacol. Res..

[2] Premoli M., Aria F., Bonini S.A., Maccarinelli G., Gianoncelli A., Pina S.D., Tambaro S., Memo M., Mastinu A. (2019). Cannabidiol: Recent advances and new insights for neuropsychiatric disorders treatment. Life Sci..

[3] Überall M.A. (2020). A Review of Scientific Evidence for THC:CBD Oromucosal Spray (Nabiximols) in the Management of Chronic Pain. J. Pain Res..

[4] Sekar K., Pack A. (2019). Epidiolex as adjunct therapy for treatment of refractory epilepsy: A comprehensive review with a focus on adverse effects. F1000Research.

[5] Huestis M.A., Solimini R., Pichini S., Pacifici R., Carlier J., Busardò F.P. (2019). Cannabidiol Adverse Effects and Toxicity. Curr. Neuropharmacol..

[6] Larsen C., Shahinas J. (2020). Dosage, Efficacy and Safety of Cannabidiol Administration in Adults: A Systematic Review of Human Trials. J. Clin. Med. Res..

[7] Carvalho R.K., Santos M.L., Souza M.R., Rocha T.L., Guimarães F.S., Anselmo-Franci J.A., Mazaro-Costa R. (2018). Chronic exposure to cannabidiol induces reproductive toxicity in male Swiss mice. J. Appl. Toxicol..

[8] Carty D.R., Thornton C., Gledhill J.H., Willett K.L. (2018). Developmental Effects of Cannabidiol and Δ9-Tetrahydrocannabinol in Zebrafish. Toxicol. Sci..

[9] ElBatsh M.M., Assareh N., Marsden C.A., Kendall D.A. (2012). Anxiogenic-like effects of chronic cannabidiol administration in rats. Psychopharmacology.

[10] Mato S., Sánchez-Gómez M.V., Matute C. (2010). Cannabidiol induces intracellular calcium elevation and cytotoxicity in oligodendrocytes. Glia.

[11] Jadoon K.A., Tan G.D., O’Sullivan S.E. (2017). A single dose of cannabidiol reduces blood pressure in healthy volunteers in a randomized crossover study. JCI Insight.

[12] Rosenkrantz H., Fleischman R.W., Grant R.J. (1981). Toxicity of short-term administration of cannabinoids to rhesus monkeys. Toxicol. Appl. Pharmacol..

[13] Ewing L.E., Skinner C.M., Quick C.M., Kennon-McGill S., McGill M.R., Walker L.A., ElSohly M.A., Gurley B.J., Koturbash I. (2019). Hepatotoxicity of a Cannabidiol-Rich Cannabis Extract in the Mouse Model. Molecules.

[14] Skinner C.M., Nookaew I., Ewing L.E., Wongsurawat T., Jenjaroenpun P., Quick C.M., Yee E.U., Piccolo B.D., ElSohly M., Walker L.A. (2020). Potential Probiotic or Trigger of Gut Inflammation—The Janus-Faced Nature of Cannabidiol-Rich Cannabis Extract. J. Diet. Suppl..

[15] Ewing L.E., McGill M.R., Yee E.U., Quick C.M., Skinner C.M., Kennon-McGill S., Clemens M., Vazquez J.H., McCullough S.S., Williams D.K. (2019). Paradoxical Patterns of Sinusoidal Obstruction Syndrome-Like Liver Injury in Aged Female CD-1 Mice Triggered by Cannabidiol-Rich Cannabis Extract and Acetaminophen Co-Administration. Molecules.

[16] Devinsky O., Nabbout R., Miller I., Laux L., Zolnowska M., Wright S., Roberts C. (2019). Long-term cannabidiol treatment in patients with Dravet syndrome: An open-label extension trial. Epilepsia.

[17] Thiele E.A., Marsh E.D., French J.A., Mazurkiewicz-Beldzinska M., Benbadis S.R., Joshi C., Lyons P.D., Taylor A., Roberts C., Sommerville K. (2018). Cannabidiol in patients with seizures associated with Lennox-Gastaut syndrome (GWPCARE4): A randomised, double-blind, placebo-controlled phase 3 trial. Lancet.

[18] Qian Y., Gurley B.J., Markowitz J.S. (2019). The Potential for Pharmacokinetic Interactions Between Cannabis Products and Conventional Medications. J. Clin. Psychopharmacol..

[19] Brunetti P., Lo Faro A.F., Pirani F., Berretta P., Pacifici R., Pichini S., Busardò F.P. (2020). Pharmacology and legal status of cannabidiol. Ann. Dell’Istituto Super. Sanita.

[20] Walker L.A., Koturbash I., Kingston R., ElSohly M.A., Yates C.R., Gurley B.J., Khan I. (2020). Cannabidiol (CBD) in Dietary Supplements: Perspectives on Science, Safety, and Potential Regulatory Approaches. J. Diet. Suppl..

[21] Laux L.C., Bebin E.M., Checketts D., Chez M., Flamini R., Marsh E.D., Miller I., Nichol K., Park Y., Segal E. (2019). Long-term safety and efficacy of cannabidiol in children and adults with treatmentresistant Lennox-Gastaut syndrome or Dravet syndrome: Expanded access program results. Epilepsy Res..

[22] Gurley B.J., Yates C.R., Markowitz J.S. (2018). “…Not Intended to Diagnose, Treat, Cure or Prevent Any Disease.” 25 Years of Botanical Dietary Supplement Research and the Lessons Learned. Clin. Pharmacol. Ther..

[23] Turton-Weeks S.M., Barone G.W., Gurley B.J., Ketel B.L., Lightfoot M.L., Abul-Ezz S.R. (2001). St John’s wort: A hidden risk for transplant patients. Prog. Transplant..

[24] Barone G.W., Gurley B.J., Ketel B.L., Lightfoot M.L., Abul-Ezz S.R. (2000). Drug interaction between St. John’s wort and cyclosporine. Ann. Pharmacother..

[25] Butawan M., Benjamin R.L., Bloomer R.J. (2017). Methylsulfonylmethane: Applications and safety of a novel dietary supplement. Nutrients.

[26] Herschler R.J. (1982). Dietary and Pharmaceutical Uses of Methyl-Sulfonylmethane and Compositions Comprising it. U.S. Patent.

[27] Herschler R.J. (1985). Methylsulfonylmethane in Dietary Products. U.S. Patent.

[28] Herschler R.J. (1986). Dietary Products and Uses Comprising Methylsulfonylmethane. U.S. Patent.

[29] Herschler R.J. (1984). Solid Pharmaceutical Compositions Comprising MSM and their Production. U.S. Patent.

[30] Debbi E.M., Agar G., Fichman G., Ziv Y.B., Kardosh R., Halperin N., Elbaz A., Beer Y., Debi R. (2011). Efficacy of methylsulfonylmethane supplementation on osteoarthritis of the knee: A randomized controlled study. BMC Complement. Altern. Med..

[31] Kim L.S., Axelrod L.J., Howard P., Buratovich N., Waters R.F. (2006). Efficacy of methylsulfonylmethane (MSM) in osteoarthritis pain of the knee: A pilot clinical trial. Osteoarthr. Cartil..

[32] Wong T., Bloomer R.J., Benjamin R.L., Buddington R.K. (2017). Small Intestinal Absorption of Methylsulfonylmethane (MSM) and Accumulation of the Sulfur Moiety in Selected Tissues of Mice. Nutrients.

[33] Gurley B.J., Murphy T.P., Gul W., Walker L.A., ElSohly M. (2020). Content versus Label Claims in Cannabidiol (CBD)-Containing Products Obtained from Commercial Outlets in the State of Mississippi. J. Diet. Suppl..

[34] Kim S.H., Smith A.J., Sanberg P.R., Tan J., Shytle R.D., Giunta B. (2015). MSM ameliorates HIV-1 tat induced neuronal oxidative stress via rebalance of the glutathione cycle. Am. J. Transl. Res..

[35] Borzellca J.F., Sipes I.G., Wallace K.B. (2007). Dossier in Support of the Generally Recognized as Safe (GRAS) Status of Optimsm (Methylsulfonylmethane; MSM) as a Food Ingredient.

[36] Maronpot R.R., Yoshizawa K., Nyska A., Harada T., Flake G., Mueller G., Singh B., Ward J.M. (2010). Hepatic enzyme induction: Histopathology. Toxicol. Pathol..

[37] Giraud C., Tran A., Rey E., Vincent J., Tréluyer J.M., Pons G. (2004). In vitro characterization of clobazam metabolism by recombinant cytochrome P450 enzymes: Importance of CYP2C19. Drug Metab. Dispos..

[38] Huddart R., Leeder J.S., Altman R.B., Klein T.E. (2018). PharmGKB summary: Clobazam pathway, pharmacokinetics. Pharmacogenet. Genomics.

[39] Magnuson B.A., Appleton J., Ames G.B. (2007). Pharmacokinetics and distribution of [35S]methylsulfonylmethane following oral administration to rats. J. Agric. Food Chem..

[40] Gerhards E., Gibian H. (1967). The metabolism of dimethyl sulfoxide and its metabolic effects in man and animals. Ann. N. Y. Acad. Sci..

[41] Otsuki S., Qian W., Ishihara A., Kabe T. (2002). Elucidation of dimethylsulfone metabolism in rat using a 35S radioisotope tracer method. Nutr. Res..

[42] Wang Q., Tang Q., Zhao L., Zhang Q., Wu Y., Hu H., Liu L., Liu X., Zhu Y., Guo A. (2020). Time serial transcriptome reveals *Cyp2c29* as a key gene in hepatocellular carcinoma development. Cancer Biol. Med..

[43] Joung Y.H., Darvin P., Kang D.Y., Sp N., Byun H.J., Lee C.-H., Lee H.K., Yang Y.M. (2016). Methylsulfonylmethane Inhibits RANKL-Induced Osteoclastogenesis in BMMs by Suppressing NF-κB and STAT3 Activities. PLoS ONE.

[44] Amirshahrokhi K., Khalili A.-R. (2017). Methylsulfonylmethane is effective against gastric mucosal injury. Eur. J. Pharmacol..

[45] Amirshahrokhi K., Bohlooli S. (2013). Effect of methylsulfonylmethane on paraquat-induced acute lung and liver injury in mice. Inflammation.

[46] Bohlooli S., Mohammadi S., Amirshahrokhi K., Mirzanejad-Asl H., Yosefi M., Mohammadi-Nei A., Chinifroush M.M. (2013). Effect of Methylsulfonylmethane Pretreatment on Aceta-minophen Induced Hepatotoxicity in Rats. Iran. J. Basic Med. Sci..

[47] Pecora F., Gualeni B., Forlino A., Superti-Furga A., Tenni R., Cetta G., Rossi A. (2006). In vivo contribution of amino acid sulfur to cartilage proteoglycan sulfation. Biochem. J..

[48] Kamel R., El Morsy E.M. (2013). Hepatoprotective effect of methylsulfonylmethane against carbon tetrachloride-induced acute liver injury in rats. Arch. Pharm. Res..

[49] Wojcikowski K., Gobe G. (2014). Animal studies on medicinal herbs: Predictability, dose conversion and potential value. Phytother. Res..

[50] US Food and Drug Administration Estimating the Maximum Safe Starting Dose in Initial Clinical Trials for Therapeutics in Adult Healthy Volunteers. https://www.fda.gov/regulatory-information/search-fda-guidance-documents/estimating-maximum-safe-starting-dose-initial-clinical-trials-therapeutics-adult-healthy-volunteers.

[51] Sousa-Lima I., Park S.-Y., Chung M., Jung H.J., Kang M.-C., Gaspar J.M., Seo J.A., Macedo M.P., Park K.S., Mantzoros C. (2016). Methylsulfonylmethane (MSM), an organosulfur compound, is effective against obesity-induced metabolic disorders in mice. Metabolism.

[52] Miousse I.R., Skinner C.M., Lin H., Ewing L.E., Kosanke S.D., Williams D.K., Avula B., Khan I.A., ElSohly M.A., Gurley B.J. (2017). Safety assessment of the dietary supplement OxyELITE^TM^ Pro (New Formula) in inbred and outbred mouse strains. Food Chem. Toxicol..

[53] Mcgill M.R., Jaeschke H. (2015). A direct comparison of methods used to measure oxidized glutathione in biological samples: 2-vinylpyridine and N-ethylmaleimide. Toxicol. Mech. Methods.

