# Supplementary material for: Safety and Molecular-Toxicological Implications of Cannabidiol-Rich Cannabis Extract and Methylsulfonylmethane Co-Administration
Source: Int J Mol Sci. 2020 Oct 21;21(20):7808. doi: 10.3390/ijms21207808 (PMC7656295; doi:10.3390/ijms21207808)
Supplement: Supplementary file 1 [file ijms-21-07808-s001.zip › Supplementary Table 1.docx]

| Supplementary Table 1. Primers used in qRT-PCR | | |
| --- | --- | --- |
| Gene | Forward | Reverse |
| *18S* | GGCGTCCCCCAACTTCTTA | GGGCATCACAGACCTGTTATTG |
| *Cyp1a2* | gacatggcctaacgtgcag | ggtcagaaagccgtggttg |
| *Cyp2b10* | aaggagaagtccaaccagca | ctctgcaacatgggggtact |
| *Cyp2c29* | ATCTGGTCGTGTTCCTAGCG | AGTAGGCTTTGAGCCCAAATAC |
| *Cyp2c65* | CCCTGTGTTCACTCTGTACTTG | AACTCCTCTTCCAGCAAACTC |
| *Cyp2c66* | CATTGAGGACCGAGTTCAAGAG | CATTGAGGACCGAGTTCAAGAG |
| *Cyp2d22* | cagtggttgtactaaatgggct | gctaggactataccttgagagcg |
| *Cyp2e1* | tccctaagtatcctccgtga | gtaatcgaagcgtttgttga |
| *Cyp3a4* | AAAGCCGCCTCGATTCTAAGC | ACTACATCCCGTGGTACAACC |
| *Cyp3a11* | acaaacaagcagggatggac | ggtagaggagcaccaagctg |
| *Mir-122* | Proprietary, Thermo-Fisher Scientific |  |
| *U6* | Proprietary, Thermo-Fisher Scientific |  |
